# Enhanced Odor Discrimination and Impaired Olfactory Memory by Spatially Controlled Switch of AMPA Receptors

**DOI:** 10.1371/journal.pbio.0030354

**Published:** 2005-10-18

**Authors:** Derya R Shimshek, Thorsten Bus, Jinhyun Kim, Andre Mihaljevic, Volker Mack, Peter H Seeburg, Rolf Sprengel, Andreas T Schaefer

**Affiliations:** **1**Max-Planck-Institut für medizinische Forschung, Heidelberg, Germany; Boston UniversityUnited States of America

## Abstract

Genetic perturbations of α-amino-3-hydroxy-5-methyl-4-isoxazolepropionate receptors (AMPARs) are widely used to dissect molecular mechanisms of sensory coding, learning, and memory. In this study, we investigated the role of Ca^2+^-permeable AMPARs in olfactory behavior. AMPAR modification was obtained by depletion of the GluR-B subunit or expression of unedited GluR-B(Q), both leading to increased Ca^2+^ permeability of AMPARs. Mice with this functional AMPAR switch, specifically in forebrain, showed enhanced olfactory discrimination and more rapid learning in a go/no-go operant conditioning task. Olfactory memory, however, was dramatically impaired. GluR-B depletion in forebrain was ectopically variable (“mosaic”) among individuals and strongly correlated with decreased olfactory memory in hippocampus and cortex. Accordingly, memory was rescued by transgenic GluR-B expression restricted to piriform cortex and hippocampus, while enhanced odor discrimination was independent of both GluR-B variability and transgenic GluR-B expression. Thus, correlated differences in behavior and levels of GluR-B expression allowed a mechanistic and spatial dissection of olfactory learning, discrimination, and memory capabilities.

## Abstract 2

Genetic manipulation of AMPA receptors in mouse forebrain dissects specific contributions of AMPA receptor expression variation in piriform cortex and hippocampus to olfactory learning and memory, not discrimination.

## Introduction

The sense of smell is of paramount importance for rodents [1], for which rapid odor discrimination and long-lasting olfactory memory permits responses to predator and prey critical for survival. Consequently, the behavioral analyses of olfactory capabilities in rodents are efficient, quantitative, and reproducible [2–4]. While in the formation and storage of olfactory memory piriform cortex [5–7], hippocampus [8,9], and olfactory bulb [10–12] are all implicated, the cellular correlates for these processes have not been clearly delineated. The contribution of the hippocampus to olfactory memory is presently controversial [2,13–18], but is deemed unlikely for simple olfactory discrimination tasks [9,19]. In fact, the most likely candidates for a cellular correlate of olfactory memory appear to be the neuronal connections in the piriform cortex due to the associational connectivity [5] and the expression of several forms of cellular and synaptic plasticity [7,20–23].

Concerning odor discrimination itself, cellular mechanisms for this process are often attributed to the inhibitory circuitry of the olfactory bulb ([24–30]; reviewed in [31–33]). Lateral inhibitory circuits were postulated, in analogy to retina [34,35], to mediate contrast enhancement [24], for which physiological recordings [24,36,37] and modeling data, based on the well-known anatomy [29], provide additional support. Such contrast enhancement may rest in large part on the particular properties of dendrodendritic synapses between the principal output neurons (mitral cells) and local inhibitory neurons (granule cells) of the olfactory bulb. In these distinct synapses, lateral and recurrent inhibition mediated by the gamma-aminobutyric acid-A system may be controlled by the activity of the closely appositioned glutamatergic part, perhaps triggering increased gamma-aminobutyric acid release by Ca^2+^ influx through glutamate-gated receptor channels ([38]; see also [39]).

Given that neuronal circuits underlying odor discrimination, as well as olfactory memory, rely on properties of fast excitatory neurotransmission mediated by α-amino-3-hydroxy-5-methyl-4-isoxazolepropionate receptors (AMPARs), we sought to alter, by genetic means, the specific functional contribution of α-amino-3-hydroxy-5-methyl-4-isoxazolepropionate (AMPA) channels containing the dominant subunit GluR-B. Of the four AMPAR constituents, GluR-A to D (GluR1 to 4), which form tetrameric channels with different binary subunit combinations, GluR-B is contained in the majority of AMPARs. GluR-B is critically involved in the formation and trafficking of AMPARs, and dominates their ion conductance and gating properties [40–46]. Notably, the normally low Ca^2+^ permeability of AMPA channels in principal neurons is solely mediated by GluR-B, due to a unique arginine residue (R587) in the functionally critical glutamine/arginine (Q/R) site of the pore-forming segment M2 [44,47,48], resulting from RNA editing of GluR-B pre-mRNA ([49]; reviewed in [50]). Hence, either GluR-B deficiency, or the expression of Q/R site-unedited GluR-B with a glutamine residue at the critical channel site, leads to increased Ca^2+^ permeability of AMPA channels, as amply demonstrated in gene-targeted mice [51,53,58,60]. Thus, the absence of GluR-B, or the expression of GluR-B(Q) in the olfactory bulb, may generate increased inhibition in mitral cells. Moreover, the ablation of GluR-B, but also changes in the extent of Q/R site editing of GluR-B, can alter the strength of excitatory synaptic transmission in the genetically addressed neuronal populations [51,58,60], thus potentially shifting the balance of excitatory and inhibitory transmission in the affected circuits. Similarly, changes in synaptic plasticity due to Ca^2+^-permeable AMPARs [51,52,60], e.g., in piriform cortex, might alter odor memorization processes. Thus, alterations of AMPAR properties in these brain regions will allow investigation and possibly separation of mechanisms underlying these behavioral traits.

Concerning this intended switch in AMPAR properties, mice lacking all GluR-B, however, show a widespread impairment in behavior, including lethargy, motor coordination problems, and deficits in exploratory activity [51], which preclude detailed behavioral analyses. Similarly, mice expressing (in the entire brain) a substantial part of the GluR-B population in the Q/R site-unedited form become seizure-prone and die prematurely [53]. Some of these problems can be partially overcome by use of spatially and temporally restricted expression systems [54–56], in particular the Cre-lox system, with Cre-recombinase expression in defined brain areas of gene-targeted mice carrying GluR-B alleles marked by loxP sites for Cre-mediated recombination [55,57]. Indeed, restricting the expression of Q/R site-unedited GluR-B to forebrain resulted in almost normal lifespan and an only weakly seizure-prone phenotype [58]. Mice with forebrain-specific GluR-B depletion appeared almost completely normal throughout life with no developmental abnormalities, thus permitting a detailed, quantitative investigation of olfactory behavior.

To allow for the mechanistic separation of olfactory learning, discrimination, and memory, we exploited a well-known phenomenon of transgenes, which concerns heterogeneous expression among different founder lines and even among genetically identical individuals of a given line. Although such “mosaic” expression is usually undesired, here we took advantage of it by ablating GluR-B via gene-targeted, floxed GluR-B alleles with the help of a transgenic mouse line with variegated Cre expression in forebrain. By correlating GluR-B levels in olfaction-related brain regions with quantitative behavioral data, we investigated the dependence on GluR-B of olfactory discrimination and memory. Moreover, to delineate the brain areas involved in these distinctive olfactory processes we used transgenic “rescue” of GluR-B ablation, specifically in piriform cortex and hippocampus.

These efforts allowed us to dissect, both spatially and mechanistically, the role of GluR-B-mediated AMPAR properties in selected brain regions in odor learning, discrimination, and memory.

## Results

### GluR-B(Q) Expression in the Forebrain Increases Olfactory Learning and Discrimination

**Figure 1:**
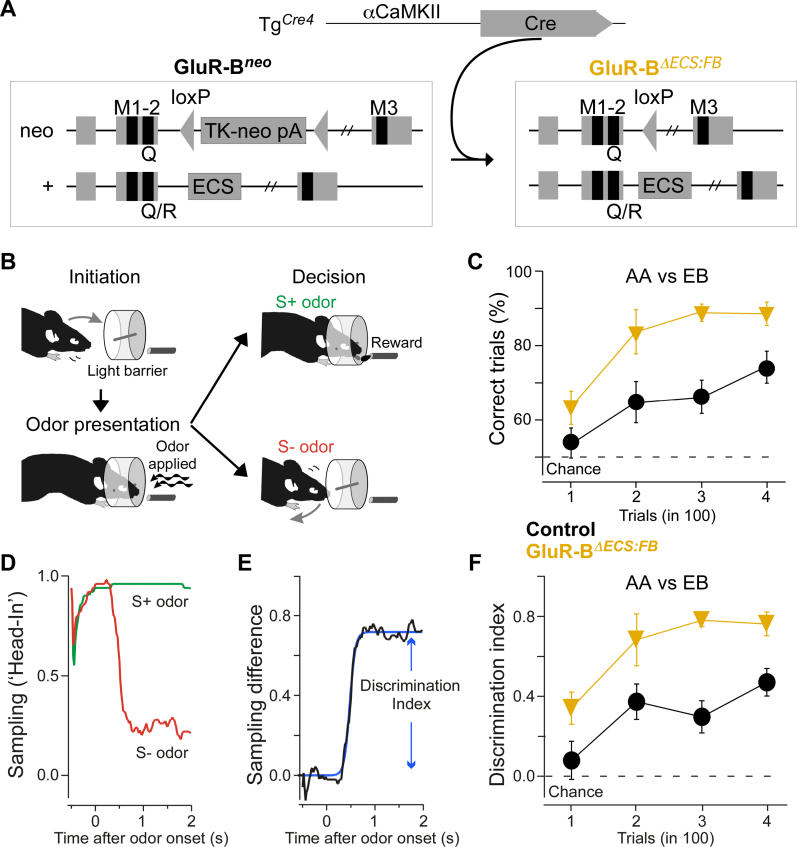
Odor Learning and Discrimination Is Enhanced in *GluR-B^ΔECS:FB^* Mice (A) Schematic diagram depicting Cre-mediated activation of GluR-B(Q) by removing the loxP-flanked TK-neo (TK-neo pA, GluR-B*^neo^*) element in intron 11, which is acting as a suppressor in expression from the Q/R site editing deficient *GluR-B^neo^* allele. Exon 10 and 11 encode membrane-spanning segments 1, 2, and 3 (M1, 2, 3) of the GluR-B subunit. The intronic element necessary for editing the Q/R site is shown for the wild-type allele (+). (B) Scheme of an individual trial. Breaking a light barrier, the mouse initiates a trial. An odor is presented, and (depending on the odor denotation and the mouse's response) the mouse is rewarded or retracts its head. A small (2- to 4-μl) water reward is given at the end of an S+ odor if the mouse continuously licks at the delivery tube during the 2-s trial. A trial is counted as correct if the mouse licks continuously upon presentation of a rewarded (S+) odor or does not lick continuously with a nonrewarded (S−) odor [3]. (C) Twelve experimentally naïve animals (six *GluR-B^ΔECS:FB^* [orange] and six *GluR-B^+/+^* littermate controls [black]) were trained on 1% AA versus 1% EB for two tasks of 200 trials each. Both groups acquired the task (> 70% correct); however, the *GluR-B^ΔECS:FB^* were both quicker, and performed better overall, than the littermate controls (group effect: F_(1,10 )_ = 10.2; *p* < 0.01). AA, amylacetate; EB, ethylbutyrate. (D) Average head position for one mouse and 50 presentations of the S+ (green) and 50 presentations of the S− (red) odor. “1” indicates the breaking of the light beam (head in the sampling port [3]). Note the rapid head retraction for the S− odor. (E) Difference of the average head positions from (D) for S+ and S− odors. Blue line indicates sigmoidal fit. “Discrimination index” refers to the maximum of the fitted sigmoid. (F) As (C) but depicting the discrimination index as a function of trial number (group effect: F_(1,10)_ = 1.7; *p* < 0.01).

To explore the role of fast excitatory neurotransmission carried by GluR-B-containing AMPA channels in olfactory processes, we first analyzed mice that express part of the GluR-B population in a Q/R site-unedited form (Figure 1A; termed “*GluR-B^ΔECS:FB^*”; see also [58]). These mice carry, in addition to a wild-type GluR-B allele, a gene-targeted *GluR-B^neo^* allele in which the intronic sequence critical for GluR-B pre-mRNA editing at the Q/R site is replaced by a floxed *TK-neo* gene, which severely reduces splicing of the modified intron and hence attenuates the expression of the *GluR-B^neo^* allele [60]. To unsilence the attenuated *GluR-B^neo^* allele, specifically in the postnatal forebrain, we crossed in the *Tg^Cre4^* transgene, which encodes Cre-recombinase and is driven by the αCaMKII promoter ([59]; “Camkcre4”). Thus, Cre-recombinase removes in forebrain neurons the intronic *TK-neo* gene, leading to the active *GluR-B^ΔECS^* allele for Q/R site-unedited GluR-B(Q) subunits (Figures 1 and S1). As expected [53,60], also in mice expressing postnatally forebrain-specific Q/R site-unedited GluR-B(Q) subunits, Ca^2+^ permeability through AMPAR was increased and AMPAR currents showed rectification ([58] and unpublished data). *GluR-B^ΔECS:FB^* mice had, in contrast to mice expressing GluR-B(Q) globally, a prolonged lifespan and no severe developmental alterations. They were however, still seizure-prone [58], and hence behavioral training was restricted to short periods of time.

We trained six *GluR-B^ΔECS:FB^* mice and six littermate controls on one odor pair in an automated go/no-go olfactory conditioning task [3,61]. In this task, water-deprived mice are trained to distinguish a water-rewarded odor (S+) and an unrewarded odor (S−) by their licking response (Figure 1B). Both *GluR-B^ΔECS:FB^* and control mice acquired the “simple” task to discriminate between the monomolecular odors amylacetate and ethylbutyrate (percentage correct > 70% after 400 trials). Strikingly, *GluR-B^ΔECS:FB^* mice showed more rapid learning and enhanced discrimination capabilities (Figure 1C, group effect: F_(1,10)_ = 10.2, *p* < 0.01). This was confirmed by fitting linear trend lines to the initial part of the learning curve (slope difference: *p* < 0.05; see Materials and Methods). The training system employed allows for careful monitoring of head positions [3]. For rewarded trials (Figure 1D, green), already weakly trained animals kept their head in the sampling port (large tube in Figure 1B) during the entire 2-s trial, whereas for unrewarded trials the head is retracted quickly (Figure 1D, red). The difference of these two curves (Figure 1E, black) is a very sensitive assay for discrimination performance; the fitted maximum of this curve (Figure 1E, blue) is referred to as the “discrimination index” in the remainder of the paper and is again strongly improved for *GluR-B^ΔECS:FB^* mice, compared with controls (Figure 1F; group effect: F_(1,10)_ = 11.7, *p* < 0.01). This was not due to general motor performance, attention, or motivation changes, as both the intertrial interval (group effect: F_(1,10)_ = 0.56, *p* > 0.4) and the overall licking frequency (group effect: F_(1,10)_ = 0.72, *p* > 0.4) were unaffected.

Thus, expression of Ca^2+^-permeable AMPARs in forebrain areas, including the olfactory bulb, resulted in more rapid odor learning and enhanced olfactory discrimination.

### *GluR-B^ΔFB^* Mice Exhibit Increased Olfactory Discrimination Performance

**Figure 2:**
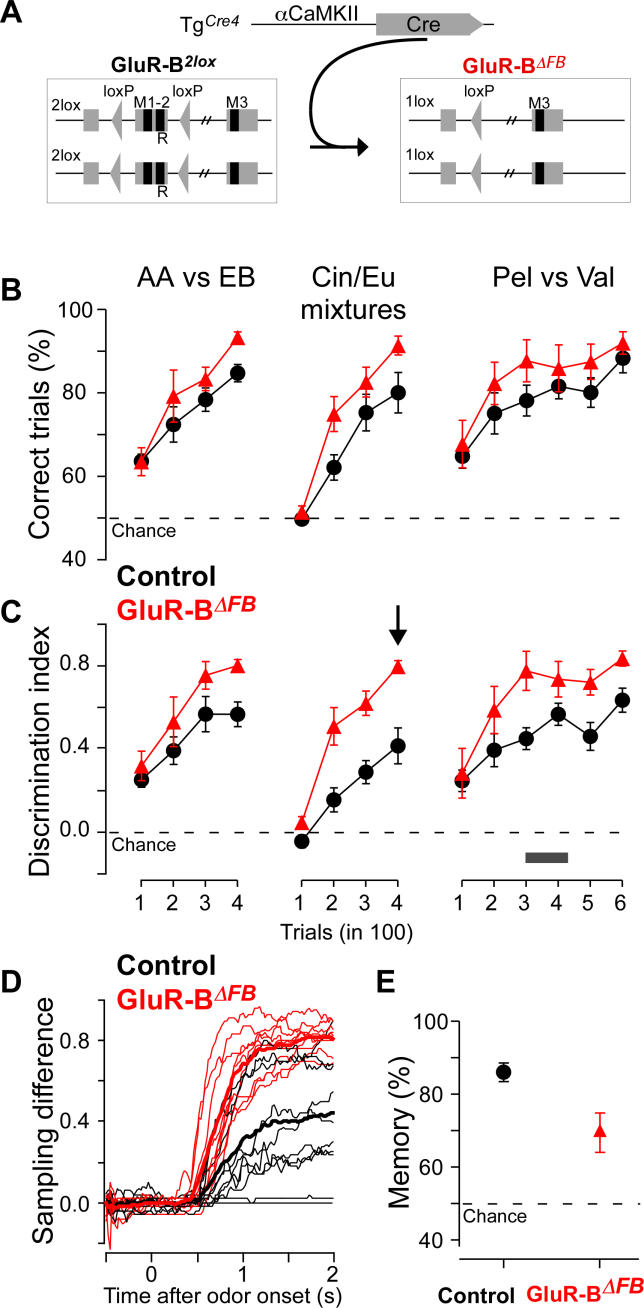
Odor Learning and Discrimination Is Enhanced, but Odor Memory Is Reduced in *GluR-B^ΔFB^* Mice (A) Schematic diagram depicting Cre-mediated ablation of loxP-flanked exon 11 of the GluR-B alleles. (B and C) Nine *GluR-B^ΔFB^* (red) and nine *GluRB^2lox^* littermate controls (black) were trained for successive odor discrimination tasks on 1% AA versus 1% EB (400 trials), 0.4% Cin/0.6% Eu versus 0.6% Cin/0.4% Eu (400 trials) and 1% Pel versus 1% Val. *GluR-B^ΔFB^* mice showed increased learning/discrimination compared with controls, both using the performance as measured by percentage of correct trials ([B]; group effect: F_1,16_ = 6.55, *p* < 0.05) or the discrimination index (C), that is the maximal difference of the sampling pattern (see Materials and Methods and Figure 1D–1F; F_1,16_ = 29.5, *p* < 10^−4^). (D) Sampling difference for the last 100 trials of the mixture discrimination task (indicated with a black arrow in [C]) for all 18 individual mice. Note that the *GluR-B^ΔFB^* mice show a consistently larger sampling difference. (E) Olfactory memory performance for nine littermate controls (black) and nine *GluR-B^ΔFB^* (red) mice. Olfactory memory was tested at the time indicated by the black bar in (C) by interleaving the Pel and Val trials with unrewarded AA and EB trials. AA, amylacetate; Cin, cineol; EB, ethylbutyrate; Eu, eugenol; Pel, pelargonic acid; Val, valeric acid.

To ascertain if enhanced olfactory learning and odor discrimination may indeed correlate with the increased Ca^2+^ permeability of AMPA channels in the Q/R site-unedited form, we next analyzed *GluR-B^ΔFB^* mice, which lack GluR-B in forebrain. This specific ablation was generated by forebrain-selective *Tg^Cre4^* expression ([59] “Camkcre4”) in gene-targeted *GluR-B^2lox^* mice carrying, in both *GluR-B* alleles, a floxed exon 11 (Figure 2A). The specific GluR-B depletion in *GluR-B^ΔFB^* mice can be monitored by immunohistochemistry (see below) and immunoblotting. In quantitative immunoblot analyses, we found GluR-B levels reduced to 28 ± 7%, 29 ± 8%, and 52 ± 9% (± SEM; *n* = 10) in the hippocampus, cortical areas, and olfactory bulb, respectively, relative to GluR-B levels in *GluR-B^2lox^* littermate controls. In the absence of GluR-B, the electrophysiological properties of AMPA channels become similar to those with GluR-B/GluR-B(Q) switch [51] showing strong rectification and increased Ca^2+^ permeability through AMPA channels (unpublished data). However, GluR-B depletion is not lethal and does not produce seizures. In addition, in contrast to the complete GluR-B knockouts, mice with forebrain-specific GluR-B depletion appeared normal throughout life with no developmental abnormalities, or difference in body size and weight in adulthood (wild-type: 31.0 g ± 1.2; *GluR-B^ΔFB^*: 28.4 ± 0.9; each *n* = 10). Exploratory activity in an open field task was slightly increased in *GluR-B^ΔFB^* mice (3,480 cm ± 180, *n* = 11), compared with wild-type littermates (2,512 cm ± 96, *n* = 12, *p* <0.01). Motor coordination measured in an accelerating rotarod was somewhat impaired in the mutant mice (wild-type: 156 s ± 37, *n* = 6; *GluR-B^ΔFB^*: 37 s ± 10, *n* = 6; *p* < 0.05). Tests in the dark/light box revealed increased anxiety of *GluR-B^ΔFB^* mice (latency of first exit: wild-type [17 s ± 3], *GluR-B^ΔFB^* [97 s ± 42], *p* = 0.047; compartment changes: wild-type [17 s ± 3], *GluR-B^ΔFB^* [7 ± 2], *p* < 0.05; time spent in lit compartment: wild-type [103 s ± 10], *GluR-B^ΔFB^* [59 s ± 19], *p* = 0.051; each *n* = 6). Hence, unlike the complete GluR-B knockouts, *GluR-B^ΔFB^* mice show only very minor changes in general activity and no sign of any major developmental disturbance, thus allowing detailed, quantitative behavioral investigations.

If the GluR-B/GluR-B(Q) switch-induced alterations in the Ca^2+^ permeability of the AMPA channels are linked to enhanced odor discrimination and learning, the depletion of the GluR-B subunit should lead to a similar phenotypic readout. We therefore trained nine *GluR-B^ΔFB^* mice and nine littermate controls in the same automated associative go/no-go olfactory conditioning task described above. To cover even small phenotypic changes in olfaction, we tested different odor pairs, “simple” monomolecular odors and “difficult” binary mixtures [3]. After habituation, *GluR-B^ΔFB^* and control mice were trained to discriminate between the “simple” monomolecular odors amylacetate and ethylbutyrate, and subsequently additionally on a “difficult” discrimination task consisting of similar binary mixtures of cineol and eugenol; and finally again on a “simple” discrimination task with the monomolecular odors pelargonic and valeric acid. Similar to *GluR-B^ΔECS:FB^, GluR-B^ΔFB^* mice also showed enhanced learning and discrimination compared with controls (Figure 2B; group effect: F_(1,16)_ = 6.55; *p* < 0.05). Increased discrimination performance is expected to show more pronounced effects for closely related odors because of the more challenging “difficult” discrimination task that is closer to the psychophysical limits of the system [3,4]. Consistent with this notion, differences between *GluR-B^ΔFB^* and control mice were not only larger if the detailed sampling pattern and discrimination index were investigated (Figure 2C and 2D, group effect: F_(1,16)_ = 29.5; *p* < 10^−4^), but in particular for the discrimination of binary mixtures with similar composition (group effect: F_(1,16)_ = 27.8; *p* < 10^−4^ for the mixture; F_AA EB(1,16)_ = 7.0; *p* = 0.02, and F_Pel Val(1,16)_ = 5.8; *p* = 0.03 for the “simple” discrimination tasks; 3-way ANOVA: F_(6,96)_ = 2.9; *p* = 0.01). As activity, measured by the intertrial interval, was not significantly different between genotypes (F_(1,16)_ = 3.1; *p* = 0.1), and analysis of the lick frequency showed a tendency to reduced motivation of *GluR-B^ΔFB^* mice in this particular task (F_(1,16)_ = 9.2; *p* < 0.01), we conclude that depletion of GluR-B in forebrain areas indeed resulted in increased olfactory learning and discrimination performances, rather than motivational alterations. To assess olfaction specificity, we trained ten *GluR-B^ΔFB^* mice and ten controls in a nonolfactory hippocampus-dependent spatial learning task (elevated Y-maze). Performance in this task was not improved compared with controls (Figure S2); on the contrary, the acquisition of this task was slightly impaired, allowing the conclusion that the enhanced olfactory discrimination performance is likely to be specific to the sense of smell and possibly related to enhanced discrimination capability, rather than a general increase in alertness. Furthermore, enhancement in odor discrimination in both *GluR-B^ΔECS:FB^* and *GluR-B^ΔFB^* mice makes it likely that improvement in this task results from the common AMPAR property change mediated by the depletion of GluR-B and the lack of the Q/R site-edited GluR-B subunit, both resulting in Ca^2+^-permeable AMPARs. Thus, this suggests that increased Ca^2+^ influx via AMPARs leads to enhanced olfactory learning and discrimination.

### Olfactory Memory Is Significantly Decreased but Highly Variable in *GluR-B^ΔFB^* Mice

To capture the full extent of the role of Ca^2+^-permeable AMPARs in olfactory behavior, we next assessed the effects of altered AMPA channels on olfactory memory. To probe olfactory memory in *GluR-B^ΔFB^* mice, six days after the end of the first training phase for odor discrimination (amylacetate versus ethylbutyrate), the training trials for the third odor pair (pelargonic versus valeric acid) were interleaved with unrewarded trials in which amylacetate or ethylbutyrate were again presented (black bar in Figure 2C). Whereas control mice reliably responded only to the previously rewarded odor (memory of 86 ± 8%, mean ± SD, *n* = 9, Figure 2E), *GluR-B^ΔFB^* mice showed reduced olfactory memory (69 ± 16%, *n* = 9, *p* < 0.05, Mann-Whitney). Due to the more rapid learning observed in *GluR-B^ΔFB^*, one could speculate that a decrease in olfactory memory might simply reflect increased extinction. However, extinction levels were low in general, no significant group-trial interaction could be found for the memory trials (2-way ANOVA, F_(6,90)_ = 1.5, *p* > 0.1), and a restriction of the analysis to early memory trials displayed essentially the same pattern (Figure S3A). Thus, reduced performance in the probe trials is not due to increased extinction but reflects genuine memory impairment. Moreover, because the hippocampus-dependent spatial memory after-task acquisition in the Y-maze was not affected (Figure S2), we conclude that the observed olfactory memory deficit is rather specific for olfaction and does not readily generalize to other modalities.

**Figure 3:**
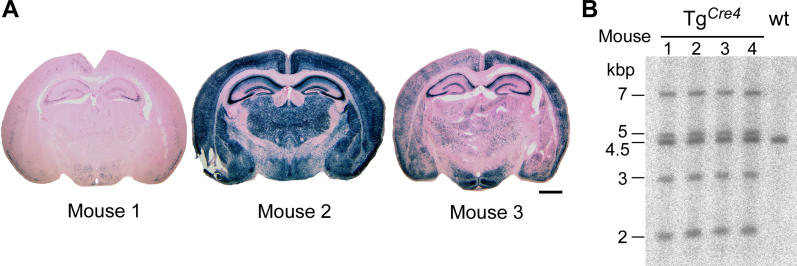
Variability of Cre Expression of Mouse Line *Tg^Cre4^* (A) Variable Cre expression in forebrains of three different mice positive for *Tg^Cre4^* and *R26R* Cre indicator (see Figure S1) at postnatal day 12 pictured by the Cre-dependent β-galactosidase activity (blue, X-gal, counterstain by eosin) in coronal brain slices. Scale bar: 1.25 mm. (B) Southern blot analysis of BglII-digested genomic mouse DNA of four *Tg^Cre4^* mice that differed in the Cre expression pattern. Southern probe detects the wild-type (4.5 kbp) and the transgenic (7, 5, 3, and 2 kbp) alleles.

While the improved odor discrimination and learning behavior showed only little variability, the significantly impaired memory performance observed in *GluR-B^ΔFB^* mice was highly variable among individual animals compared with control littermates (Figure 2E). This variability in olfactory memory was reflected in the level and extent of Cre-recombinase expression in forebrain of transgenic *Tg^Cre4^* mice, as visualized by Cre-activity in the Cre-indicator mouse line *R26R*. We observed that onset and extent of Cre-recombinase expression in different forebrain regions varied among individual *Tg^Cre4^* mice (Figure 3A), which could also be directly visualized by immunohistochemistry with a Cre-antibody (unpublished data). As this variability persisted after several backcrosses, and Southern blot analysis revealed no differences of transgene integration or number among animals (Figure 3B), it could not be attributed to genetic differences but rather to epigenetic mechanisms.

Hence, we hypothesized that the variability in olfactory memory reflected the mosaicism observed in the transgenic *Tg^Cre4^* line. The evaluation of regional differences in expression pattern of animals with robust and poor olfactory memory could then be applied to identify brain areas responsible for the observed phenotypes.

### Olfactory Memory Correlates with Residual GluR-B Protein Expression in *GluR-B^ΔFB^* Mice

**Figure 4:**
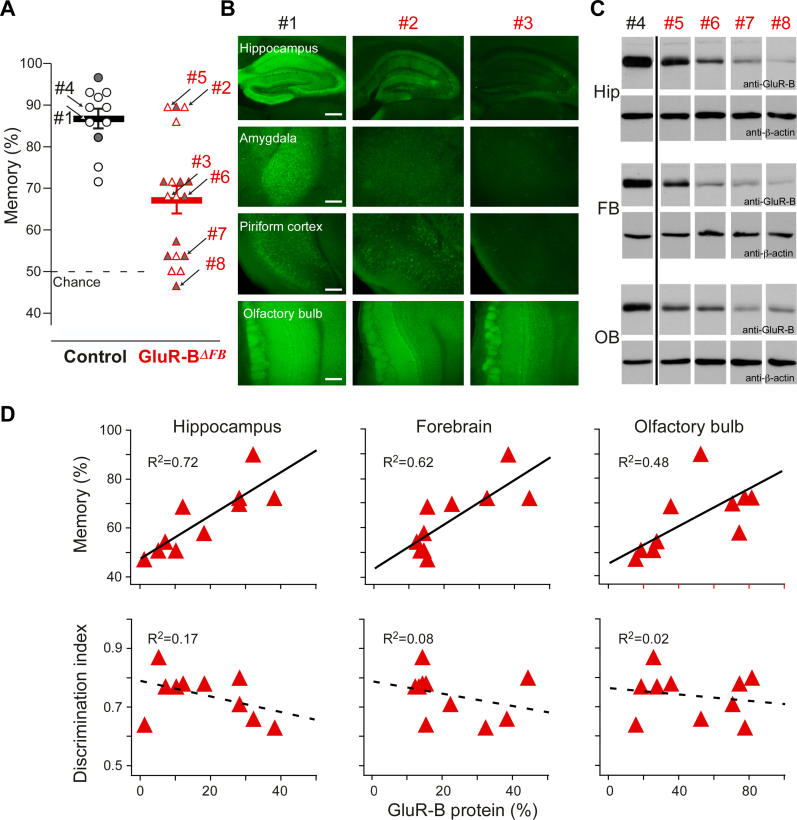
Olfactory Memory but not Odor Learning/Discrimination Is Correlated with Residual GluR-B Levels in Hippocampus and Forebrain of *GluR-B^ΔFB^* Mice (A) The olfactory memory performance for 18 *GluR-B^ΔFB^* (red) and 11 littermate control (black) mice is given as mean (thick lines ± SEM) and as individual performance in open circles and triangles. Arrows with numbers (#) indicate those mice used in experiments (B–C). Data were combined from Figure 2 (open symbols) and an additional experiment with nine *GluR-B^ΔFB^* and two littermate controls (shaded symbols). (B and C) Residual GluR-B levels as detected by anti-GluR-B immunofluorescence in hippocampus, amygdala, piriform cortex, and olfactory bulb of one control (#1) and two *GluR-B^ΔFB^* (#2 and #3) coronal mouse brain sections (B) and by immunoblot analysis from hippocampal (Hip), cortical forebrain (FB), and olfactory bulb (OB) protein extracts of control (#4) and *GluR-B^ΔFB^* mice (#5, #6, #7, and #8) probed with antibodies detecting GluR-B and β-actin as an internal loading control (C). Scale bars: 200 μm (first panel), 100 μm (other panels). (D) From ten *GluR-B^ΔFB^* mice, the individual odor learning/discrimination and olfactory memory performance was determined together with the relative GluR-B levels in immunoblots of hippocampal, forebrain, and olfactory bulb protein extracts. Memory performance (top panels) and discrimination capability (bottom panels; discrimination index is measured for the last 100 trials of the mixture discrimination task as indicated by the arrow in Figure 2C) were plotted against GluR-B levels. Memory was tightly correlated to GluR-B protein level in hippocampus (R^2^ = 0.72; *p* < 0.003) and cortical forebrain (R^2^ = 0.62; *p* < 0.006) and only weakly in the olfactory bulb (R^2^ = 0.48; *p* = 0.03). No measure of learning/discrimination (discrimination index for last 100 mixture trials [D], slopes of trend lines, average discrimination index, average sampling pattern differences, correct performance, etc. [not shown]) displayed any correlation (R^2^ < 0.3).

Thus, to examine whether the pronounced variability of olfactory memory in *GluR-B^ΔFB^* mice (Figure 2E) reflects variability of GluR-B levels in *GluR-B^ΔFB^* mice, we analyzed the residual amount of GluR-B protein in mice with disparate memory performances (Figure 4A–C). Notably, mice with pronounced memory deficits (memory < 70%) showed essentially no detectable GluR-B protein in hippocampus, amygdala, olfactory bulb, and piriform cortex (*n* = 2, Figure 4B, and unpublished data), but mice with almost complete memory displayed substantial residual GluR-B levels in all brain areas investigated (*n* = 2, Figure 4B, and unpublished data).

To quantify the relation between residual GluR-B protein and olfactory memory, the memory experiment was repeated with nine additional *GluR-B^ΔFB^* mice and two *GluR-B^2lox^* control animals (indicated with shaded symbols in Figure 4A), resulting in the same mean, variability, and range of memory performance (control: 89 ± 10%; *GluR-B^ΔFB^*: 63 ± 14%). Subsequently, protein was extracted from olfactory bulbs, cortical areas, and hippocampi from each mouse, and GluR-B protein was quantified (Figure 4C). The summarized correlations are depicted in Figure 4D (two animals were used for immunofluorescent analysis that yielded the same results as in the first experiment). Whereas no learning or discrimination-related parameter correlated with residual protein levels (Figure 4D, R^2^ < 0.3), a strong correlation between memory and GluR-B protein was observed in hippocampus (Figure 4D, R^2^ = 0.72, *p* < 0.003, *n* = 10) and cortical areas (Figure 4D, R^2^ = 0.62, *p* < 0.006, *n* = 10). Only a weakly significant correlation was found in the olfactory bulb (Figure 4D, R^2^ = 0.48, *p* = 0.03, *n* = 10). GluR-A levels were unchanged from wild-type, indicating that compensatory up-regulation of other AMPAR subunits is unlikely (GluR-A levels relative to control: 1.02 ± 0.05, mean ± SEM, *n* = 10).

In summary, mice with reduced GluR-B levels in forebrain areas showed decreased olfactory memory, which correlated tightly with a reduction in GluR-B levels in the hippocampus and cortical areas. Enhanced odor learning and discrimination, on the other hand, was independent of residual GluR-B levels in the olfactory bulb and other forebrain areas, indicating that moderate GluR-B reductions are sufficient to saturate enhanced odor learning and discrimination. Thus, although both are mediated by alterations in the AMPAR subunit GluR-B, due to the qualitatively different dose-response curves, the phenotypes regarding olfactory memory, and olfactory learning/discrimination must be mechanistically distinct.

### Partial Rescue of Olfactory Memory Deficit by Selective Transgenic GluR-B Expression in Hippocampus and Piriform Cortex in *GluR-B^ΔFB^* Mice

The effect of selective GluR-B depletion in mice indicated that GluR-B-containing AMPARs in the hippocampus, and/or (olfactory) cortex are likely to be important for olfactory memory. The olfactory memory phenotype could be due to depletion of GluR-B in olfactory cortex or hippocampus; enhanced learning and discrimination capabilities might rather be evoked by AMPARs lacking GluR-B in the olfactory bulb.

**Figure 5:**
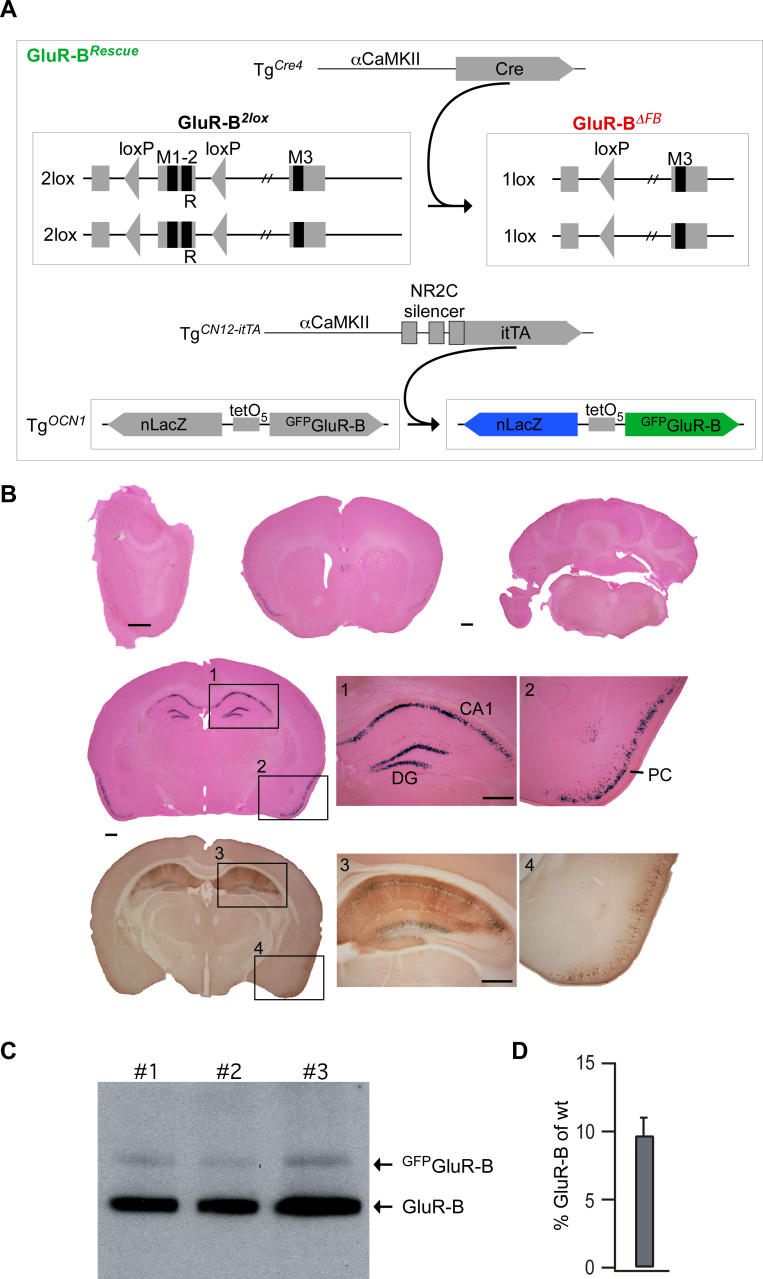
Specific Hippocampus and Piriform Cortex Expression of Transgenic ^GFP^GluR-B (A) Schematic diagrams depicting forebrain-specific GluR-B deletion as in Figure 2A and itTA-dependent expression of ^GFP^GluR-B and nuclear-localized β-galactosidase (nLacZ) in *GluR-B^ΔFB^* mice (termed *GluR-B^Rescue^*). ^GFP^GluR-B and nLacZ are both encoded by *Tg^OCN1^,* and itTA is controlled by a fusion of the NR2C silencer element [91] and the αCaMKII promoter (termed *Tg^CN12-itTA^* [92]). (B) In coronal brain sections of mice positive for both transgenes *(Tg^CN12-itTA^* and *Tg^OCN1^)* β-galactosidase activity (blue, X-gal, counterstain by eosin) is restricted to hippocampal neurons in CA1, DG, and neurons in the piriform cortex. The same neurons show ^GFP^GluR-B expression when analyzed in immunohistochemical sections with an antibody against GFP. Scale bars: 500 μm. (C) Immunoblot detecting endogenous GluR-B and transgenic ^GFP^GluR-B in the hippocampus of three different mice (#1, #2, #3). (D) Relative quantification from (C) of transgenic ^GFP^GluR-B compared with endogenous GluR-B in the hippocampus.

To obtain independent evidence for this spatial and mechanistic dissection of the roles of Ca^2+^-permeable AMPARs, we expressed by transgenic means N-terminally green fluorescent protein (GFP)-tagged GluR-B specifically in hippocampus and piriform cortex of *GluR-B^ΔFB^* mice (Figure 5A and 5B). In accordance with the proposed region-dependence, we expected that additional GluR-B subunits in hippocampus and/or piriform cortex improve odor memory but do not alter odor discrimination performance. The mouse line employed for this purpose, termed *GluR-B^Rescue^*, had the genetic background of *GluR-B^ΔFB^* mice but additionally carried a bidirectional module for β-galactosidase and ^GFP^GluR-B expression, responsive to the tetracycline-controlled transcriptional transactivator [62]. The transactivator was under the control of a modified αCaMKII-promoter fragment to obtain high expression selectivity (Figure 5A; see also Materials and Methods). The transgenic expression level of ^GFP^GluR-B was 9.72% ± 1.25 (*n* = 3) in the hippocampus, compared with endogenous GluR-B (Figure 5C and 5D). Analysis of β-galactosidase activity and ^GFP^GluR-B expression in brain sections of *GluR-B^Rescue^* mice revealed expression in hippocampus and piriform cortex, whereas cortex, amygdala, and striatum only rarely showed any positive cells (Figure 5B). Importantly, both the spatial pattern and intensity of ^GFP^GluR-B expression were constant among all *GluR-B^Rescue^* mice analyzed (*n* = 11).

**Figure 6:**
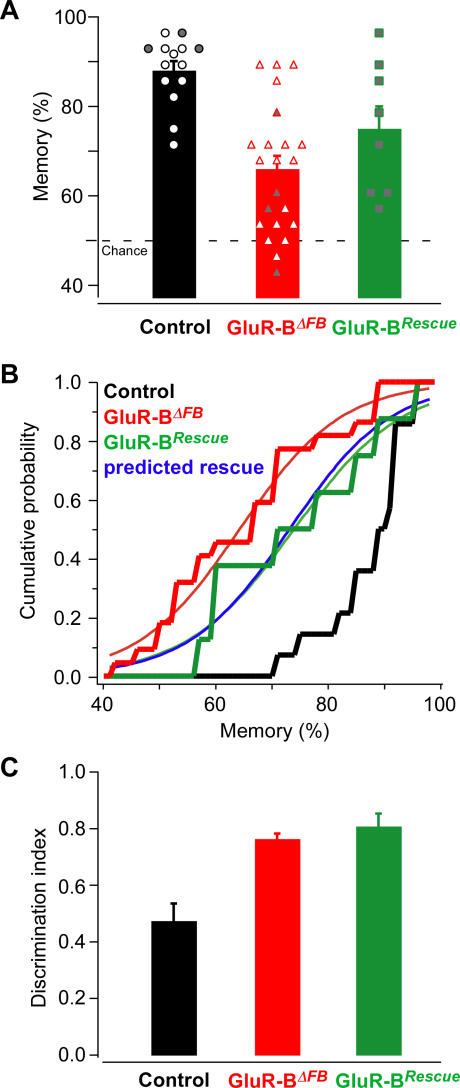
GluR-B Expression in Hippocampus and Forebrain Partially Rescues the Memory Deficit of *GluR-B^ΔFB^* Mice (A) Olfactory memory experiments as in Figure 2 were performed with *GluR-B^Rescue^*. Individual mice are indicated: control *GluR-B^2lox^* with black circles, *GluR-B^ΔFB^* with red triangles, *GluR-B^Rescue^* with green squares. Data were combined from Figure 3 and 4 (open symbols) and an additional experiment with four *GluR-B^ΔFB^*, three littermate controls, and eight *GluR-B^Rescue^* (shaded symbols). (B) Cumulative histogram of the memory performance. Memory performance is indicated as a stepped line. The sigmoidal fit is indicated as a continuous line. The predicted rescue on the basis of the extent of transgenic ^GFP^GluR-B expression (Figure 5D) and the correlation between memory and GluR-B levels in piriform cortex (9.1 ± 2.5% increase in memory for 10% increase in protein, Figure 4D) is shown in blue. Note that the predicted rescue is in perfect numerical correspondence to the memory performance of *GluR-B^Rescue^* mice. (C) The discrimination index was calculated as in Figures 1E, 2C, and 4D (last 100 trials of the mixture discrimination task) for *GluRB^Rescue^* (*n* = 8), *GluR-B^ΔFB^* (*n* = 22), and control (*n* = 14) animals.

Olfactory memory experiments with *GluR-B^Rescue^* , and both *GluR-B^ΔFB^* and *GluR-B^2lox^* mice as controls, were performed as described above (indicated with shaded symbols in Figure 6A). Memory was again highly reproducible in both *GluR-B^ΔFB^* (66 ± 12%; *n* = 4) and *GluR-B^2lox^* (94 ± 2%; *n* = 3) mice, compared with experiments performed earlier (Figures 2E and 4A). Importantly, olfactory memory in *GluR-B^Rescue^* mice was intermediate (75 ± 15%, *n* = 8), below *GluR-B^2lox^* control levels, but better than in *GluR-B^ΔFB^* mice. Assessing memory under extinction-free condition, where each trial was rewarded, confirmed again that the memory deficit was a true memory deficit and not due to increased extinction (Figure S3B). Data from the experiments described in Figures 2 and 4 were combined to allow statistical comparison (Figure 6A and 6B). In summary, *GluR-B^Rescue^* mice showed both enhanced memory performances compared with *GluR-B^ΔFB^* (overall ANOVA: F_(2,41)_ = 13.6, *p* < 10^−4^; memory_Rescue_ = 75 ± 15%, *n* = 8; memory_ΔFB_ = 66 ± 14%, *n* = 22; *p* < 0.05; Figure 6A), but were still impaired relative to *GluR-B^2lox^* controls (memory_2lox_ = 88 ± 8%, *n* = 14; *p* < 0.005), consistent with a partial rescue of the memory deficit by circumscribed transgenic ^GFP^GluR-B expression in hippocampus and piriform cortex. Notably, the partial memory is in numerical agreement with the predictions from the protein correlation and the measurement of transgenic protein expression (see predicted memory, blue line, in Figure 6B). In the olfactory memory experiments with *GluR-B^ΔFB^* animals, olfactory memory linearly depended on GluR-B expression in cortex and hippocampus with a slope of 9.1 ± 2.5% (cortex) and 8.9 ± 2.0% (hippocampus) increase in memory per 10% increase in protein (Figure 4D). A 9.7% increase in GluR-B in these brain regions, as achieved by *GluR-B^Rescue^* animals (Figure 5), is thus predicted to increase olfactory memory by approximately 9% throughout the heterogeneous population (blue line in Figure 6B). This confirms the role of these brain areas as inferred from the mosaic expression and protein correlation analysis described above (Figure 4). However, odor discrimination (measured by the discrimination index as in Figures 1, 2, and 4D) was as enhanced as in *GluR-B^ΔFB^* mice (0.79 ± 0.05, mean ± SEM compared with 0.76 ± 0.02, *p* > 0.7, Figure 6C), and improved relative to *GluR-B^2lox^* controls (0.48 ± 0.06; Figure 6C; overall ANOVA: F_(2,41)_ = 17.2, *p* < 10^−5^; post hoc Newman Keuls: *p* < 10^−3^), as expected if the enhanced discrimination phenotype is due to Ca^2+^-permeable AMPARs in the olfactory bulb and unaffected by GluR-B expression in piriform cortex or hippocampus.

Hence, transgenic GluR-B expression, specifically in the piriform cortex and hippocampus in the GluR-B knockout background, rescues the odor memory deficit but leaves enhanced olfactory discrimination and learning unaltered.

## Discussion

Here we present mechanistic and spatial dissections of olfactory discrimination, learning, and memory. We employed gene-targeted and transgenic mice with region-specific expression to demonstrate that a change in GluR-B-mediated properties of AMPA channels in αCaMKII-expressing neurons of mouse forebrain, including olfactory bulb mitral and granule cells, enhances olfactory discrimination and learning but impairs olfactory memory. These pertinent olfactory behaviors were assessed in a go/no-go operant conditioning task, which provides a quantitative, robust, and reproducible behavioral tool [3]. We observed among individual mice a striking variability in olfactory memory performance but not in odor discrimination. This variability could be traced to epigenetic variability in the transgenic expression of Cre-recombinase, which mediated recombination within loxP-flanked segments of gene-targeted alleles for the dominant AMPAR subunit GluR-B, and hence operated the switch in AMPAR properties toward GluR-B ablation and increased Ca^2+^ permeability. In contrast to variable memory, olfactory discrimination and learning performances appeared already saturated by even moderate extents of Cre expression, and hence moderate changes in AMPAR properties. The subsequent transgene-driven re-introduction of GluR-B, specifically in piriform cortex and hippocampus, reversed the Cre-induced loss of GluR-B and partially rescued the odor memory deficit, but left unaltered the enhanced olfactory discrimination. In a nutshell, we conclude that olfactory discrimination is enhanced by an increase in AMPAR-mediated Ca^2+^ permeability within the olfactory bulb, whereas olfactory memory becomes impaired upon genetically induced GluR-B ablation in higher brain centers, specifically in piriform cortex.

### Olfactory Discrimination Is Increased in Mice with Forebrain-Specific GluR-B Ablation or GluR-B(Q) Expression

Both forebrain-specific GluR-B(Q) expression and GluR-B depletion led to increased olfactory learning and discrimination capabilities. This was rather pronounced for GluR-B(Q)-expressing mice, consistent with the overall stronger phenotypic consequences in comparison to GluR-B depletion, both when forebrain-selective ([58], and this study) or global [53,60].

A detailed analysis of the sampling pattern [3], and in particular the analysis of discrimination tasks that involved “simple” dissimilar monomolecular odor pairs and “difficult” binary mixtures, were necessary to fully capture the characteristics of the olfactory discrimination phenotype for forebrain-specific GluR-B-depleted mice. For closely related binary mixtures, discrimination improvements were largest, consistent with a *specific* alteration in odor discrimination, rather than a general enhancement of learning capabilities. This is further supported by the notion that no general improvement was observed in other nonolfactory behavioral tasks, e.g., hippocampus-dependent spatial learning tasks such as matching-to-place spatial reference memory tasks (Figure S2). As no Cre expression and activity was observed in the main olfactory sensory neurons at any developmental stage (Figure S4 and unpublished data), olfactory epithelial function was unaltered by the genetic modification. The vomeronasal organ showed very weak Cre expression (unpublished data), but a role of this structure concerning performance in the olfactory discrimination task is unlikely (for review, see [63]). The behavioral phenotype is thus likely to be associated with the *processing* of olfactory information rather than the detection of odors. Furthermore, increased performance even after long stretches of training and, in particular, increased sampling pattern differences (TB and ATS, unpublished data) supported the notion that GluR-B depletion resulted in enhanced odor *discrimination* capability. The learning phenotype might thus be a result of this enhanced discrimination capability or reflect additional changes in circuits underlying task acquisition.

### Putative Cellular Basis of Enhanced Odor Discrimination and Learning

AMPAR properties were altered specifically in neurons of forebrain areas, most notably olfactory bulb, olfactory cortex, and other cortical areas and hippocampus, leading to enhanced odor discrimination and learning. Interestingly, transgenic expression of ^GFP^GluR-B in *GluR-B^ΔFB^* mice (genetic “rescue”), specifically in piriform cortex and hippocampus with no detectable expression in the olfactory bulb, did not alter enhanced discrimination and learning capabilities. This is consistent with a primary role of the olfactory bulb in olfactory discrimination and learning. Alternatively, transgenic protein levels might have been too low to alter discrimination and learning capabilities, although memory was clearly affected. This notion will be further tested in mice with piriform cortex-specific GluR-B ablation.

A direct contribution to the phenotype by Ca^2+^ influx through genetically modified AMPA channels seems to be likely, since mice expressing Q/R site-unedited GluR-B showed even better odor learning and discrimination performance compared with mice with depleted GluR-B. In both mouse models Ca^2+^ influx through AMPA channels is increased, whereas the effect on the macroscopic AMPA conductance differs. AMPAR currents are reduced in mice not expressing GluR-B [51], possibly due to fewer synaptic AMPA channels and impaired AMPAR trafficking and recycling. In mice expressing the unedited form of GluR-B(Q), macroscopic conductance in whole-cell patches of CA1 pyramidals is increased [60], but excitatory transmission in CA3-to-CA1 cell synapses is somewhat reduced, in spite of a lower threshold for generating a population spike [58], indicating increased synaptic excitability by sustained GluR-B(Q) expression, in line with the seizure-prone phenotype. Yet, both mouse models yield enhanced discrimination and learning capability. The milder phenotype upon GluR-B depletion could be attributed to reduced AMPAR densities at synapses [40,41] and therefore probably to a lesser extent of Ca^2+^ influx. This is further supported by kainate-induced Co^2+^ uptake in acute brain slices, revealing considerably higher Co^2+^ uptake via Ca^2+^-permeable AMPARs in hippocampal pyramidal neurons of mice expressing GluR-B(Q), than those lacking GluR-B (DRS, RS, and PHS, unpublished data).

Normally, GluR-B-containing AMPARs are prominently localized at the dendrodendritic synapse between mitral and granule cells in the olfactory bulb [64,65]. As Ca^2+^ influx through glutamate receptors is thought to contribute to lateral and recurrent inhibition ([38] see also [39]), the absence of GluR-B, or the presence of GluR-B(Q) in the olfactory bulb, is likely to result in increased inhibition between the principal neurons in this structure. Notably, the improved olfactory discrimination capabilities were apparent even upon relatively small reductions in GluR-B levels. It will be interesting to see if *GluR-B^+/neo^* mice also exhibit enhanced odor discrimination, which is indeed likely given that low levels of GluR-B(Q), and hence a small increase in Ca^2+^-permeable AMPARs, arise from the attenuated *GluR-B^neo^* allele [60], and, moreover, that GluR-B levels are decreased in these mice due to the single copy of the *GluR-B^+^* allele.

In virtually all models of the olfactory bulb, lateral (and, in fewer models, also recurrent) inhibition plays a dominant role, either in establishing spatiotemporal dynamics [32,66–69], or in directly enhancing contrast and therefore simplifying discrimination and learning of similar odorants [24,28,29,36]. Increased inhibition will thus in general improve discriminability, consistent with the behavioral improvements observed with GluR-B(Q) expression, the GluR-B knockout, and the “rescue” mice. A direct test of this link would require the quantitative measurement of inhibition in the intact preparation, a task that might be feasible with further improved in vivo electrophysiological techniques, such as targeted recordings [70,71] or simultaneous pre- and postsynaptic intracellular measurements.

### Olfactory Memory Is Reduced in GluR-B Knockout Mice and Improved by GluR-B Expression in Piriform Cortex and Hippocampus

To assess olfactory memory of *GluR-B^ΔFB^* mice, after six days the mice were probed with unrewarded odor presentations that interleaved a simple discrimination task. Prolonged behavioral tasks such as the assessment of long-term olfactory memory were not performed with the seizure-prone *GluR-B^ΔECS:FB^* mice. Memory in *GluR-B^ΔFB^* mice, however, was dramatically impaired. This cannot be attributed to a general, unspecific deficit because, simultaneous to the memory trials, the normal, rewarded discrimination task was performed even better than by controls. Reduced olfactory memory can also not be simply attributed to increased extinction, as no significant trial-group interactions were observed and also, when restricted to the first unrewarded memory trials, a significant impairment was observed (Figure S3A). Additionally, investigating relearning of the first discrimination task revealed a significant correlation with the memory performance, showing a “memory” deficit under extinction-free conditions (Figure S3B). Finally, in other memory-related tasks, such as a hippocampus-dependent spatial reference memory task (Figure S2), mice with GluR-B depletion were not impaired after task acquisition. We therefore conclude that forebrain-specific ablation of GluR-B results in a specific loss of long-term olfactory memory but, at the same time, in enhanced odor discrimination and learning capabilities.

To dissect the discrimination, learning, and memory phenotypes, and ultimately identify potential cellular correlates, we made use of the variegated Cre expression of *Tg^Cre4^* mice. Therefore, residual GluR-B levels were determined from each mouse that had been tested in the behavioral experiments, and levels were correlated with odor learning, discrimination, and olfactory memory. The significant direct correlation between residual GluR-B levels in hippocampus and cortical areas with olfactory memory suggested hippocampal and/or cortical neurons as putative mediators for odor memorization and/or storage. No measure of discrimination and learning, on the other hand, correlated with residual GluR-B levels; even the smallest reduction in GluR-B levels (about 20%–60%) was sufficient to establish and saturate enhanced odor discrimination capabilities. Together, this allows the conclusion that distinct mechanisms mediate discrimination/learning and memory. This is furthermore consistent with a prominent role of cortical areas or hippocampus in olfactory memory, although GluR-B levels in the olfactory bulb were also weakly correlated with memory performance. Transgenic expression of ^GFP^GluR-B in piriform cortex and hippocampus indeed rescued memory to an extent strikingly consistent with the transgenic protein levels (approximately 10% of wild-type GluR-B), confirming the reliability of the correlation analysis and the sensitivity of the behavioral assay.

In general, the hippocampus is thought to be involved in only those olfactory memory tasks that involve temporal sequence analysis or require other higher cognitive features [2,13,72–74]. In particular, extensive lesions to the hippocampal formation in rats do not interfere with long-term olfactory memory in go/no-go successive olfactory discrimination tasks [72], such as the one described. This leaves piriform cortex as the most prominent candidate for the locus of the olfactory memory deficit described, consistent with the prevalent view of piriform cortex as an associational memory structure [75] and learning-associated changes in piriform cortex [7,20–23].

### Potential Mechanisms of Specific Olfactory Memory Impairment

What could be the cellular basis of the long-term memory deficit brought about by lack of GluR-B-containing AMPARs? For complete GluR-B knockouts, increased long-term plasticity (LTP) was reported in hippocampal field recordings [51]. In addition, in hippocampal and amygdala pathways lacking GluR-B, an AMPAR-dependent, *N*-methyl-D-aspartate (NMDA) receptor-independent form of LTP can be readily induced [51,52]. Mechanistically, this is likely to be due to Ca^2+^ influx through GluR-B-less AMPA channels leading to non-hebbian forms of plasticity. However, hebbian dependence on simultaneous pre- and postsynaptic activity is often a critical feature for memory storage (reviewed in e.g. [76]); hence, a non-hebbian form of plasticity should result in impaired memorization.

Surprisingly, in *GluR-B^ΔFB^* mice no LTP changes could be observed in field measurements in the hippocampus, nor could NMDA-independent LTP be induced in hippocampal synapses between Schaffer collaterals and CA1 pyramidal cells in presence of the NMDA antagonist APV (K. Jensen, O. Hvalby, personal communication). However, in other brain areas that are potentially important for the processing of olfactory information, such as piriform cortex or olfactory bulb, LTP measurements have not yet been performed; synapses of these pathways may be regulated differently than hippocampal CA3/CA1 synapses.

Additionally, by changing the Ca^2+^ permeability of AMPARs, Ca^2+^ signaling via AMPA and colocalized NMDA channels might be disturbed, thereby impairing memory formation. Other forms of plasticity [77,78] induced by Ca^2+^-permeable AMPARs might play a prevalent role in olfactory memory. Alternatively, long-term stabilization might involve GluR-B phosphorylation similar to cerebellar long-term depression [79] and thus be selectively impaired by GluR-B depletion. Physiological experiments to assess these hypotheses will ideally make use of even more restricted genetic modifications with completely undisturbed input structures. One possibility would be Cre-mediated GluR-B depletion and GluR-B(Q) expression in piriform cortex as suggested above.

### Correlating Quantitative Behavior and Mosaic Gene Expression for Dissecting Phenotypes

A critical step in discerning the discrimination and learning phenotypes from the memory phenotype was the observation of increased variability in the memory of the GluR-B knockout animals and, subsequently, the individual analyses of protein expression levels. We made use of the epigenetic variability in GluR-B excision that could be correlated to the variability in memory but showed no correlation to variability in discrimination.

The analysis of quantitative trait loci (for review, see [80]), or classically correlating different behavioral phenotypes within a truly wild-type population [81–84], also attempts to find common or distinct genetic origins of different behavioral traits. These attempts undoubtedly contribute significantly to unraveling the molecular basis of behavior. Due to subtle and multigenic differences between different strains of rodents or different individuals in a wild-type population, however, they suffer from a rather low “signal-to-noise” ratio. Differences in individual genes are rather small compared with the vast number of genes involved. As a result, purely correlating behavioral traits within a wild-type population usually remains rather descriptive, whereas quantitative trait loci analysis is capable of revealing multigenic basis of behavioral traits but usually lacks the power to identify the individual genes themselves.

Herein, we described a way to enhance the signal-to-noise ratio by making use of the mosaic expression often associated with transgenic approaches: combining the advantages of “classical” genetic manipulation—namely, that the target of the manipulation is well defined—with the possibility to analyze variability, might also in future provide novel ways to define molecular and cellular correlates of complex behavioral traits.

In this study, we combined genetically induced manipulation of the AMPAR composition with quantitative behavioral and molecular analyses. We could thus provide evidence for opposing roles of specific GluR-B manipulation and increased Ca^2+^ influx via AMPARs in olfactory discrimination/learning, and memory, potentially in the olfactory bulb and piriform cortex, respectively. To achieve this, we made use of the epigenetic variability in the extent of GluR-B ablation, which correlated with the variability in odor memory, but not in discrimination and learning; this finding was subsequently confirmed in GluR-B rescue mice with piriform cortex-specific transgenic expression of GluR-B. Extending these principles of combining quantitative behavioral analyses with minimal and dosed genetic interference, together with further physiological analyses, will ultimately pinpoint the neural circuitries underlying related, but distinct, behavioral traits in olfactory and other systems.

## Materials and Methods

### Mouse lines

The ***R26R*** line [85] was employed as Cre indicator. ***GluR-B*** *^Δ**ECS:FB**^* mice were of mixed C57Bl/6 and NMRI genetic background and generated from ***Tg^Cre4^*** (“Camkcre4” in [59]) and *GluR-B^+/neo^* [60] mice. ***GluR-B^+/neo^*** mice carry a wild-type GluR-B allele and a gene-targeted GluR-B allele in which the intron 11 sequence critical for Q/R site editing is replaced by a *TK-neo* gene flanked by loxP sites (“floxed”). ***GluR-B^2lox^*** mice [86] carry gene-targeted GluR-B alleles in which exon 11 is floxed. ***GluR-B*** *^Δ**FB**^* mice were of C57Bl/6 genetic background and generated from *Tg^Cre4^* and *GluR-B^2lox^* mice. ***Tg^CN12-itTA^*** mice were generated as described for *Tg^CN10-itTA^* mice (see Materials and Methods in [58]), and represent another founder line obtained from the same pronucleus injection, with more widespread forebrain expression. For exogeneous expression of ^GFP^GluR-B, the mouse line ***Tg^OCN1^*** was generated: an AseI fragment of plasmid pnlacZ/^GFP^GluR-B was injected into the pronucleus of oocytes obtained from DBA1/C57Bl/6 F1 hybrids. Positive founders were backcrossed into C57Bl/6 for further analysis. Plasmid pnlacZ/^GFP^GluR-B was constructed from pnlacZ/^GFP^GluR-A [87] by replacing GluR-A cDNA with the rat cDNA for GluR-B. Transgenic ^GFP^GluR-B protein levels were measured in hippocampus of *Tg^OCN1^* mice also carrying a transgene for forebrain-specific homogeneous tTA expression [88].

### Experimental groups

Mice heterozygous for *Tg^Cre4^* and heterozygous for the TK-neo cassette in the GluR-B allele *(GluR-B^ΔECS:FB^)* or homozygous for the floxed GluR-B *(GluR-B^ΔFB^)* were used in the experiments. The “rescue” mice (***GluR-B^Rescue^***) were positive for *Tg^Cre4^,* homozygous for the floxed GluR-B gene, and positive for the tet-sensitive responder transgene *Tg^OCN1^* and for the itTA expressing activator transgene *Tg^CN12-itTA^*.

### Control groups

Littermate controls were used in all experiments. *GluR-B^+/+^* mice were used as controls in the task described in Figure 1. GluR-B*^+/2lox^* and GluR-B*^2lox/2lox^* (both negative for *Tg^Cre4^*) mice were used as controls in experiments described in Figures 2, 4, and 6. For the *GluR-B^Rescue^* experiments (Figure 6), controls positive for either *Tg^CN12-itTA^* or *Tg^OCN1^* in a GluR-B*^+/2lox^* and GluR-B*^2lox/2lox^* (both negative for *Tg^Cre4^*) background were used.

### Genotyping

Mice were selected by PCR of mouse-tail DNA with specific primers as described below. Indicated are the sequences and the approximate length of the amplified DNA fragments. *Tg^Cre4^*: rspCre1 (5′- ACCAGGTTCGTTCACTCATGG-3′) and rspCre2 (5′- AGGCTAAGTGCCTTCTCTACAC-3′), 200 basepairs (bp).

*GluR-B^neo^*: MH60 (5′- CACTCACAGCAATGAAGCAGGAC-3′), MH53a (5′- GAATGTTGATCATGTGTTTCCCTG-3′), and MH117 (5′- GTTCGAATTCGCCAATGACAAGACG-3′), wild-type: 500 bp and mutant: 400 bp.

*GluR-B^2lox^*: VM12 (5′- GCGTAAGCCTGTGA AATACCTG-3′) and VM10 (5′- GTTGTCTAACAAGTTGTTGACC-3′), wild-type: 250 bp and mutant: 350 bp.

*Tg^OCN1^*: VM4 (5′- CTCCCAGACAACCATTACCTGTCC-3′) and GluR-B882BST (5′- CGAAGTATACTTAATTGTCGCTGTGTG-3′), 600 bp.

*Tg^CN12-itTA^*: htTA1 (5′- AGAGCAAAGTCATCAACTCTG-3′) and htTA2 (5′- GTGAGAGCCAGACTCACATTTCA-3′), 1,000 bp.

### Southern blot analysis

Genomic DNA from mouse-tail/liver was digested with restriction enzyme BglII (NEB), and the Southern blot was done with a 320-bp probe (“integ”) obtained by PCR detecting the αCaMKII promoter.

### Histochemistry

Histochemistry was performed as described previously [89], with the following exceptions: Coronal 70- to 100-μm vibratome slices were used for immunohistochemistry with GluR-B (1:60, polyclonal; Chemicon, Temecula, California, United States), GFP (1:8,000, polyclonal; MobiTech, Göttingen, Germany), and Cre (1:8,000, polyclonal; BAbCO, Berkeley, California, United States) primary antibodies, and FITC-coupled (1:200; Dianova, Hamburg, Germany) and peroxidase-coupled (1:600; Vector, Burlingame, California, United States) secondary goat anti-rabbit antibodies.

The main olfactory epithelium was obtained via cryostat sectioning, and immunohistochemistry was performed (primary antibody Cre, 1:5,000, polyclonal; BAbCO). X-gal staining was performed as described [89].

### Immunoblot analysis

Mouse brains were removed, and the hippocampus, olfactory bulb, and remaining forebrain areas were isolated. Total protein was prepared, and immunoblots were performed as described [87]. Antibodies used were against GluR-B (1:800, monoclonal; Chemicon), β-actin (1:40,000, monoclonal; Sigma, St. Louis, Missouri, United States) as an internal standard, and secondary goat anti-rabbit and goat anti-mouse antibodies (Vector, 1:15,000). Immunoreactivity was detected with ECLplus (Amersham, Little Chalfont, United Kingdom), and immunoblots were scanned and quantitatively analyzed with ImageJ.

### Behavioral analysis: Subjects

All mice were four to six weeks old at the beginning of the experiments. Subjects were maintained on a 12-h light-dark cycle in isolated cages in a temperature and humidity-controlled animal facility. All behavioral training was conducted during daytime. During the training period, animals were kept on free food but on a water-restriction schedule designed to keep them at > 85% of their free food body weight. Continuous water restriction was never longer than 12 h. All animal care and procedures were in accordance with the animal ethics guidelines of the Max-Planck Society.

### Apparatus

All olfactory discrimination experiments were performed using three modified eight- channel olfactometers ([61], Knosys, Bethesda, Maryland, United States of America) operated by custom-written software in Igor (Wave Metrics, Lake Oswego, Oregon, United States of America) on Pentium I, II, and III PCs running Microsoft Windows 98. Great care was taken to counterbalance groups between setups. In brief, animals were presented with odor from one out of eight possible odor channels and rewarded with a 2- to 4-μl drop of water in a combined odor/reward port (Figure 1B), ensuring tight association of the water-reward with a presented odorant. Head insertion into the port was monitored by an IR beam and photodiode (Figure 1B). Odors used were n-amyl acetate, ethyl butyrate, pelargonic acid, valeric acid, and binary mixtures of cineol and eugenol. If not otherwise noted, odors were diluted to 1% in mineral oil (Fluka Chemie, Steinheim, Germany) and further diluted by airflow to a final concentration of approximately 0.15%. All dilutions in the text refer to the dilution in mineral oil. All chemicals were obtained from Fluka Chemie.

### Task habituation training

Beginning 1–3 d after the start of the water restriction schedule, animals were trained using standard operant-conditioning procedures [3]. In a first pretraining step, each lick at the water delivery tube was rewarded. After 20 licks, a second stage was entered in which head insertion initiated a 2-s “odor” presentation during which a lick was rewarded. The “odorant” used in the pretraining was the carrier medium mineral oil. All animals learned this task within one day (2–3 sessions 30 min each).

### Structure of an individual trial

The mouse initiates each trial by breaking a light barrier at the opening of the sampling port (see also [3]). This opens one of eight odor valves, and a diversion valve that diverts all air flow away from the animal for typically 500 ms. After the release of the diversion valve, the odor is accessible to the animal for 2,000 ms. If it continuously licks at the lick port during this time (once in at least three out of four 500-ms bins), it can receive a 2- to 4-μl water reward after the end of the 2,000-ms period. If the animal does not continuously lick, or if the presented odor was a designated nonrewarded odor, neither a reward is given nor any sort of punishment, to minimize stress for the animal. Trials are counted as correct if the animal licks continuously upon presentation of a rewarded odor or does not lick continuously with a nonrewarded odor. A second trial cannot be initiated unless an intertrial interval of at least 5 s has passed. This interval is sufficiently long so that animals typically retract quickly after the end of the trial. Odors are presented in a pseudo-randomized scheme (no more than two successive presentations of the same odor, equal numbers within each 20-trial block). No intrinsic preference toward any of the odors was observed but controlled for by counterbalancing.

A total of 100–300 trials were performed each day, separated into 30- to 40-min stretches to ensure maximal motivation despite the mild water restriction scheme. Additionally, motivation was controlled by monitoring intertrial intervals and the response frequency [3].

### Measurement of performance

The simplest measure of performance is the fraction of trials in which the animal responds correctly—that is, responds with licking to the presentation of the S+ odor and does not lick with presentation of the S− odor.

It was shown previously, however, that the detailed sampling pattern is a more sensitive measure of discrimination performance [3]. To avoid long (> 3-week) training periods, we chose not to measure discrimination times [3] but to analyze the average sampling behavior in total. Upon presentation of a rewarded odor, the animal usually continuously breaks the beam, whereas upon presentation of an unrewarded odor the head is quickly retracted. The difference in response to the rewarded and unrewarded odor is approximately sigmoidal (Figures 1D, 1E, and 2D) and yields a sensitive measure of the discrimination performance. From this difference or from a sigmoidal fit to the difference (Figure 1E), several measures of discrimination can be determined: the average difference, peak, or maximum, time of half maximum, and slope of the fitted sigmoid. Whereas for small trial numbers (< 200) the slope often is not well constrained, any of the other parameters yielded essentially the same results. The discrimination index plotted in Figures 1F, 2C, 4D, and 6C refers to the fitted maximum, generally ranging from zero to one, one indicating the best discrimination. Identical results were obtained with other measures of discrimination, such as the average sampling difference.

### Structure of training

After habituation, mice were trained to discriminate 1% amylacetate from 1% ethylbutyrate for 500 trials. During the last 100 trials, the S+ odor was rewarded in only 50% of the cases to increase the resistance to extinction of the acquired memory. These trials were excluded for the statistical analysis of the learning curves. Inclusion did not alter the result of the ANOVA; however, linear fitting of the learning curve was not appropriate anymore as partial saturation of learning performance had already occurred. Subsequently, animals were trained for 500 trials on the “difficult” discrimination task [3] between the binary mixtures 0.6% eugenol/0.4% cineol and 0.4% eugenol/0.6% cineol. To allow comparison, the last 100 trials were altered as for the “simple” discrimination task above. After two days of rest, animals were finally trained on the “simple” discrimination task between 1% pelargonic acid and 1% valeric acid for another 600 trials.

In all experiments, counterbalancing between both odors and setups was ensured within and between genetic groups, or results were compared with counterbalanced subgroups, which in every case yielded identical results. During the entire course of the experiment, the person handling the animals and operating the olfactometers was blind to the genotype of the mice.

### Memory measurement

To assess memory, after 280 trials of training to discriminate between pelargonic acid and valeric acid, memory trials were interleaved for 120 trials; that is, within each block of 20 trials two unrewarded amylacetate and two unrewarded ethylbutyrate trials were included. Memory scores are given as the fraction of those unrewarded trials that were responded to “correctly” (licking response to the odor that was rewarded in the initial training session [S+], no response to the odor that was not rewarded initially [S−]). Due to the epileptic phenotype and the slightly increased mortality [58], *GluRB^ΔECS:FB^* mice were trained only for the initial period of 400 trials.

### Statistics

Learning curves for both correct performance (“percentage correct”) and the discrimination performance were analyzed by repeated measure ANOVA. Additionally, learning curves were assessed by linearly fitting of trend lines to the data with fixed offsets, leaving the slope as the only variable. In general, binning was 100 trials per block. To allow for the investigation of group/block interactions, the repeated measure ANOVA binning was reduced to 20 trials per block. To compare memory performance in the *GluR-B^Rescue^* (*n* = 8) and *GluR-B^ΔFB^* (*n* = 22) mice, due to the high variability, a bootstrap approach was employed. Subpopulations of eight animals were selected from the population of 22 *GluR-B^ΔFB^* mice, and mean memory was determined. In only 343 out of 20,000 subpopulations, mean memory exceeded the mean *GluR-B^Rescue^* memory of 74.99%, resulting in a *p* value of *p* = 343/20,000 = 0.017.

## Supporting Information

Figure S1 Forebrain-Specific Gene Manipulation (A) Schematic diagrams depicting Cre under the αCaMKII promoter control [59] and Cre-dependent expression of β-galactosidase (LacZ) by the *R26R* indicator mouse [85].
(B) Coronal forebrain sections (left) and sagittal brainstem/cerebellum sections of mice at P42 positive for *Tg^Cre4^* and *R26R* Cre-indicator. Cre expression visualized by enzymatic β-galactosidase activity (blue, X-gal, counterstain by eosin) of *R26R* and by anti-Cre immunostainings (brown, DAB [diaminobenzadine]) was restricted to forebrain regions. Scale bars: 1 mm. A, amygdala; Ce, Cerebellum; Cx, cortex; H, hippocampus; Me, medulla oblongata.
(C) Olfactory bulb sections of the same mouse to visualize Cre expression by Cre immunoreactivity (left, DAB) and by enzymatic β-galactosidase activity (right, X-gal, counterstain by eosin) in granule cells (GC) and mitral cells (MC) as indicated by arrows. Scale bars: 400 μm (upper panel), 50 μm (lower panel).
(3.4 MB TIF).

Figure S2 Performance of *GluR-B^ΔFB^* (*n* = 10) and Littermate Control (*n* = 10) Mice in a Spatial Reference Task on the Elevated Y-Maze Details of the methodology are described in [90]. * indicates *p* < 0.05.
(126 KB TIF).

Figure S3 Memory Deficit Is Not Due to Increased Extinction (A) Memory performance as a function of time for the experiment described in Figure 2E (nine *GluR-B^ΔFB^* and nine *GluR-B^2lox^* control animals). Only four unrewarded “memory +” trials are binned for each data point. Memory of *GluR-B^ΔFB^* animals was significantly reduced (F_(1,33)_ = 17; *p* < 0.001). Whereas a weak overall time effect could be observed (F_(2,66)_ = 3.6; *p* = 0.03), there was no genotype-time interaction effect (F_(2,66)_ = 0.67; *p* = 0.5), indicating that there is no differential effect of putative extinction on memory performance. * indicates *p*-values for a Mann-Whitney U test (* < 0.05, ** < 0.01).
(B) After the memory experiments from Figure 6A, the last set of animals (eight *GluR-B^Rescue^,* four *GluR-B^ΔFB^,* and three controls) was further trained on AA versus EB for 900 trials one week after the memory experiment. Subsequently, training continued on AA/EB mixtures for 1,200 trials. Finally, the animals were retrained on AA and EB for 100 trials. Relearning performance during the last retraining task is highly correlated to the original memory performance (R^2^ = 0.46, *p* = 0.006, *n* = 15).
(264 KB TIF).

Figure S4 Absence of Cre Expression in the Main Olfactory Epithelium of *Tg^Cre4^* Mice (A) Overview of the main olfactory epithelium. (B) Higher magnification of (A) showing all cells with propidium iodide (red) and absence of Cre-positive cells (green, anti-Cre). (C) Positive control showing granule cells of the dentate gyrus stained for nuclear Cre (green) from the same mouse.
(3.7 MB TIF).

## Abbreviations

AMPA: α-amino-3-hydroxy-5-methyl-4-isoxazolepropionate
AMPAR: α-amino-3-hydroxy-5-methyl-4-isoxazolepropionate receptor
bp: basepair
GFP: green fluorescent protein
LTP: long-term plasticity
NMDA: *N*-methyl-D-aspartate
Q: glutamine
R: arginine

## References

[pbio-0030354-b01] Doty RL (1976). Mammalian olfaction, reproductive processes, and behavior.

[pbio-0030354-b02] Slotnick B (2001). Animal cognition and the rat olfactory system. Trends Cogn Sci.

[pbio-0030354-b03] Abraham NM, Spors H, Carleton A, Margrie TW, Kuner T (2004). Maintaining accuracy at the expense of speed; stimulus similarity defines odor discrimination time in mice. Neuron.

[pbio-0030354-b04] Uchida N, Mainen ZF (2003). Speed and accuracy of olfactory discrimination in the rat. Nat Neurosci.

[pbio-0030354-b05] Haberly LB, Bower JM (1989). Olfactory cortex: Model circuit for study of associative memory?. Trends Neurosci.

[pbio-0030354-b06] Barkai E, Hasselmo MH (1997). Acetylcholine and associative memory in the piriform cortex. Mol Neurobiol.

[pbio-0030354-b07] Quinlan EM, Lebel D, Brosh I, Barkai E (2004). A molecular mechanism for stabilization of learning-induced synaptic modifications. Neuron.

[pbio-0030354-b08] Wood ER, Dudchenko PA, Eichenbaum H (1999). The global record of memory in hippocampal neuronal activity. Nature.

[pbio-0030354-b09] Fortin NJ, Agster KL, Eichenbaum HB (2002). Critical role of the hippocampus in memory for sequences of events. Nat Neurosci.

[pbio-0030354-b10] Brennan P, Kaba H, Keverne EB (1990). Olfactory recognition: A simple memory system. Science.

[pbio-0030354-b11] Wilson DA, Sullivan RM (1994). Neurobiology of associative learning in the neonate: Early olfactory learning. Behav Neural Biol.

[pbio-0030354-b12] Yuan Q, Harley CW, Darby-King A, Neve RL, McLean JH (2003). Early odor preference learning in the rat: Bidirectional effects of cAMP response element-binding protein (CREB) and mutant CREB support a causal role for phosphorylated CREB. J Neurosci.

[pbio-0030354-b13] Slotnick BM, Stebbins W, Berkeley M (1990). Olfactory perception. Comparative perception.

[pbio-0030354-b14] Dudchenko PA, Wood ER, Eichenbaum H (2000). Neurotoxic hippocampal lesions have no effect on odor span and little effect on odor recognition memory but produce significant impairments on spatial span, recognition, and alternation. J Neurosci.

[pbio-0030354-b15] Alvarez P, Lipton PA, Melrose R, Eichenbaum H (2001). Differential effects of damage within the hippocampal region on memory for a natural, nonspatial odor-odor association. Learn Mem.

[pbio-0030354-b16] Kaut KP, Bunsey MD, Riccio DC (2003). Olfactory learning and memory impairments following lesions to the hippocampus and perirhinal-entorhinal cortex. Behav Neurosci.

[pbio-0030354-b17] Winocur G (1990). Anterograde and retrograde amnesia in rats with dorsal hippocampal or dorsomedial thalamic lesions. Behav Brain Res.

[pbio-0030354-b18] Burton S, Murphy D, Qureshi U, Sutton P, O'Keefe J (2000). Combined lesions of hippocampus and subiculum do not produce deficits in a nonspatial social olfactory memory task. J Neurosci.

[pbio-0030354-b19] Kesner RP, Gilbert PE, Barua LA (2002). The role of the hippocampus in memory for the temporal order of a sequence of odors. Behav Neurosci.

[pbio-0030354-b20] Saar D, Grossman Y, Barkai E (1999). Reduced synaptic facilitation between pyramidal neurons in the piriform cortex after odor learning. J Neurosci.

[pbio-0030354-b21] Saar D, Grossman Y, Barkai E (2002). Learning-induced enhancement of postsynaptic potentials in pyramidal neurons. J Neurophysiol.

[pbio-0030354-b22] Roman FS, Chaillan FA, Soumireu-Mourat B (1993). Long-term potentiation in rat piriform cortex following discrimination learning. Brain Res.

[pbio-0030354-b23] Litaudon P, Mouly AM, Sullivan R, Gervais R, Cattarelli M (1997). Learning-induced changes in rat piriform cortex activity mapped using multisite recording with voltage sensitive dye. Eur J Neurosci.

[pbio-0030354-b24] Yokoi M, Mori K, Nakanishi S (1995). Refinement of odor molecule tuning by dendrodendritic synaptic inhibition in the olfactory bulb. Proc Natl Acad Sci U S A.

[pbio-0030354-b25] Urban NN, Sakmann B (2002). Reciprocal intraglomerular excitation and intra- and interglomerular lateral inhibition between mouse olfactory bulb mitral cells. J Physiol.

[pbio-0030354-b26] Margrie TW, Sakmann B, Urban NN (2001). Action potential propagation in mitral cell lateral dendrites is decremental and controls recurrent and lateral inhibition in the mammalian olfactory bulb. Proc Natl Acad Sci U S A.

[pbio-0030354-b27] Stopfer M, Bhagavan S, Smith BH, Laurent G (1997). Impaired odour discrimination on desynchronization of odour-encoding neural assemblies. Nature.

[pbio-0030354-b28] Margrie TW, Schaefer AT (2003). Theta oscillation coupled spike latencies yield computational vigour in a mammalian sensory system. J Physiol.

[pbio-0030354-b29] Linster C, Cleland TA (2004). Configurational and elemental odor mixture perception can arise from local inhibition. J Comput Neurosci.

[pbio-0030354-b30] Rospars JP, Fort JC (1994). Coding of odour quality: Roles of convergence and inhibition. Network.

[pbio-0030354-b31] Urban NN (2002). Lateral inhibition in the olfactory bulb and in olfaction. Physiol Behav.

[pbio-0030354-b32] Laurent G (1999). A systems perspective on early olfactory coding. Science.

[pbio-0030354-b33] Mori K, Nagao H, Yoshihara Y (1999). The olfactory bulb: Coding and processing of odor molecule information. Science.

[pbio-0030354-b34] DeVries SH, Baylor DA (1993). Synaptic circuitry of the retina and olfactory bulb. Cell.

[pbio-0030354-b35] Mach E (1865). Über die Wirkung der räumlichen Vertheilung des Lichtreizes auf die Netzhaut. Sitzungsberichte der Akademie der Wissenschaften in Wien Mathematisch-Naturwissenschaftliche Klasse.

[pbio-0030354-b36] Katoh K, Koshimoto H, Tani A, Mori K (1993). Coding of odor molecules by mitral/tufted cells in rabbit olfactory bulb. II. Aromatic compounds. J Neurophysiol.

[pbio-0030354-b37] Mori K, Yoshihara Y (1995). Molecular recognition and olfactory processing in the mammalian olfactory system. Prog Neurobiol.

[pbio-0030354-b38] Chen WR, Xiong W, Shepherd GM (2000). Analysis of relations between NMDA receptors and GABA release at olfactory bulb reciprocal synapses. Neuron.

[pbio-0030354-b39] Isaacson JS (2001). Mechanisms governing dendritic gamma-aminobutyric acid (GABA) release in the rat olfactory bulb. Proc Natl Acad Sci U S A.

[pbio-0030354-b40] Petralia RS, Sans N, Wang YX, Vissel B, Chang K (2004). Loss of GLUR2 alpha-amino-3-hydroxy-5-methyl-4-isoxazoleproprionic acid receptor subunit differentially affects remaining synaptic glutamate receptors in cerebellum and cochlear nuclei. Eur J Neurosci.

[pbio-0030354-b41] Sans N, Vissel B, Petralia RS, Wang YX, Chang K (2003). Aberrant formation of glutamate receptor complexes in hippocampal neurons of mice lacking the GluR2 AMPA receptor subunit. J Neurosci.

[pbio-0030354-b42] Seidenman KJ, Steinberg JP, Huganir R, Malinow R (2003). Glutamate receptor subunit 2 Serine 880 phosphorylation modulates synaptic transmission and mediates plasticity in CA1 pyramidal cells. J Neurosci.

[pbio-0030354-b43] Shi S, Hayashi Y, Esteban JA, Malinow R (2001). Subunit-specific rules governing AMPA receptor trafficking to synapses in hippocampal pyramidal neurons. Cell.

[pbio-0030354-b44] Geiger JR, Melcher T, Koh DS, Sakmann B, Seeburg PH (1995). Relative abundance of subunit mRNAs determines gating and Ca2+ permeability of AMPA receptors in principal neurons and interneurons in rat CNS. Neuron.

[pbio-0030354-b45] Greger IH, Khatri L, Ziff EB (2002). RNA editing at arg607 controls AMPA receptor exit from the endoplasmic reticulum. Neuron.

[pbio-0030354-b46] Greger IH, Khatri L, Kong X, Ziff EB (2003). AMPA receptor tetramerization is mediated by Q/R editing. Neuron.

[pbio-0030354-b47] Hume RI, Dingledine R, Heinemann SF (1991). Identification of a site in glutamate receptor subunits that controls calcium permeability. Science.

[pbio-0030354-b48] Burnashev N, Monyer H, Seeburg PH, Sakmann B (1992). Divalent ion permeability of AMPA receptor channels is dominated by the edited form of a single subunit. Neuron.

[pbio-0030354-b49] Sommer B, Kohler M, Sprengel R, Seeburg PH (1991). RNA editing in brain controls a determinant of ion flow in glutamate-gated channels. Cell.

[pbio-0030354-b50] Seeburg PH, Higuchi M, Sprengel R (1998). RNA editing of brain glutamate receptor channels: Mechanism and physiology. Brain Res Brain Res Rev.

[pbio-0030354-b51] Jia Z, Agopyan N, Miu P, Xiong Z, Henderson J (1996). Enhanced LTP in mice deficient in the AMPA receptor GluR2. Neuron.

[pbio-0030354-b52] Mahanty NK, Sah P (1998). Calcium-permeable AMPA receptors mediate long-term potentiation in interneurons in the amygdala. Nature.

[pbio-0030354-b53] Brusa R, Zimmermann F, Koh DS, Feldmeyer D, Gass P (1995). Early-onset epilepsy and postnatal lethality associated with an editing-deficient GluR-B allele in mice. Science.

[pbio-0030354-b54] Tsien JZ, Huerta PT, Tonegawa S (1996). The essential role of hippocampal CA1 NMDA receptor-dependent synaptic plasticity in spatial memory. Cell.

[pbio-0030354-b55] Tsien JZ, Chen DF, Gerber D, Tom C, Mercer EH (1996). Subregion- and cell type-restricted gene knockout in mouse brain. Cell.

[pbio-0030354-b56] Nakazawa K, Quirk MC, Chitwood RA, Watanabe M, Yeckel MF (2002). Requirement for hippocampal CA3 NMDA receptors in associative memory recall. Science.

[pbio-0030354-b57] Sauer B, Henderson N (1989). Cre-stimulated recombination at loxP-containing DNA sequences placed into the mammalian genome. Nucleic Acids Res.

[pbio-0030354-b58] Krestel HE, Shimshek DR, Jensen V, Nevian T, Kim J (2004). A genetic switch for epilepsy in adult mice. J Neurosci.

[pbio-0030354-b59] Mantamadiotis T, Lemberger T, Bleckmann SC, Kern H, Kretz O (2002). Disruption of CREB function in brain leads to neurodegeneration. Nat Genet.

[pbio-0030354-b60] Feldmeyer D, Kask K, Brusa R, Kornau HC, Kolhekar R (1999). Neurological dysfunctions in mice expressing different levels of the Q/R site-unedited AMPAR subunit GluR-B. Nat Neurosci.

[pbio-0030354-b61] Bodyak N, Slotnick B (1999). Performance of mice in an automated olfactometer: Odor detection, discrimination and odor memory. Chem Senses.

[pbio-0030354-b62] Gossen M, Bujard H (2002). Studying gene function in eukaryotes by conditional gene inactivation. Annu Rev Genet.

[pbio-0030354-b63] Firestein S (2001). How the olfactory system makes sense of scents. Nature.

[pbio-0030354-b64] Montague AA, Greer CA (1999). Differential distribution of ionotropic glutamate receptor subunits in the rat olfactory bulb. J Comp Neurol.

[pbio-0030354-b65] Sassoe-Pognetto M, Ottersen OP (2000). Organization of ionotropic glutamate receptors at dendrodendritic synapses in the rat olfactory bulb. J Neurosci.

[pbio-0030354-b66] Bazhenov M, Stopfer M, Rabinovich M, Abarbanel HD, Sejnowski TJ (2001). Model of cellular and network mechanisms for odor-evoked temporal patterning in the locust antennal lobe. Neuron.

[pbio-0030354-b67] Laurent G, Stopfer M, Friedrich RW, Rabinovich MI, Volkovskii A (2001). Odor encoding as an active, dynamical process: Experiments, computation, and theory. Annu Rev Neurosci.

[pbio-0030354-b68] Rabinovich M, Volkovskii A, Lecanda P, Huerta R, Abarbanel HD (2001). Dynamical encoding by networks of competing neuron groups: Winnerless competition. Phys Rev Lett.

[pbio-0030354-b69] Fdez GalánR, Sachse S, Galizia CG, Herz AV (2004). Odor-driven attractor dynamics in the antennal lobe allow for simple and rapid olfactory pattern classification. Neural Comput.

[pbio-0030354-b70] Margrie TW, Meyer AH, Caputi A, Monyer H, Hasan MT (2003). Targeted whole-cell recordings in the mammalian brain in vivo. Neuron.

[pbio-0030354-b71] Luo M, Katz LC (2001). Response correlation maps of neurons in the mammalian olfactory bulb. Neuron.

[pbio-0030354-b72] Otto T, Schottler F, Staubli U, Eichenbaum H, Lynch G (1991). Hippocampus and olfactory discrimination learning: Effects of entorhinal cortex lesions on olfactory learning and memory in a successive-cue, go-no-go task. Behav Neurosci.

[pbio-0030354-b73] Eichenbaum H, Fagan A, Cohen NJ (1986). Normal olfactory discrimination learning set and facilitation of reversal learning after medial-temporal damage in rats: Implications for an account of preserved learning abilities in amnesia. J Neurosci.

[pbio-0030354-b74] Eichenbaum H, Fagan A, Mathews P, Cohen NJ (1988). Hippocampal system dysfunction and odor discrimination learning in rats: Impairment or facilitation depending on representational demands. Behav Neurosci.

[pbio-0030354-b75] Wilson M, Shepherd GM, Arbib MA (1998). Olfactory cortex. Handbook of brain theory and neural networks.

[pbio-0030354-b76] Arbib MA (1998). Handbook of brain theory and neural networks.

[pbio-0030354-b77] Hoffman DA, Sprengel R, Sakmann B (2002). Molecular dissection of hippocampal theta-burst pairing potentiation. Proc Natl Acad Sci U S A.

[pbio-0030354-b78] Jensen V, Kaiser KM, Borchardt T, Adelmann G, Rozov A (2003). A juvenile form of postsynaptic hippocampal long-term potentiation in mice deficient for the AMPA receptor subunit GluR-A. J Physiol.

[pbio-0030354-b79] Chung HJ, Steinberg JP, Huganir RL, Linden DJ (2003). Requirement of AMPA receptor GluR2 phosphorylation for cerebellar long-term depression. Science.

[pbio-0030354-b80] Mackay TF (2001). The genetic architecture of quantitative traits. Annu Rev Genet.

[pbio-0030354-b81] Matzel LD, Han YR, Grossman H, Karnik MS, Patel D (2003). Individual differences in the expression of a “general” learning ability in mice. J Neurosci.

[pbio-0030354-b82] Henry KR, Buckholt NS, Bowman RE, Bovet D, Bovetnit F (1969). Genetics of memory. Science.

[pbio-0030354-b83] Hirsch J (1963). Behavior genetics and individuality understood. Science.

[pbio-0030354-b84] McGuire TR, Hirsch J (1977). Behavior-genetic analysis of Phormia regina: Conditioning, reliable individual differences, and selection. Proc Natl Acad Sci U S A.

[pbio-0030354-b85] Soriano P (1999). Generalized lacZ expression with the ROSA26 Cre reporter strain. Nat Genet.

[pbio-0030354-b86] Mack V (2001). Regulation hippocampaler synaptischer Plastizität durch konditionale Expression der AMPA-Rezeptor Untereinheit GluR-A [dissertation].

[pbio-0030354-b87] Mack V, Burnashev N, Kaiser KM, Rozov A, Jensen V (2001). Conditional restoration of hippocampal synaptic potentiation in Glur-A-deficient mice. Science.

[pbio-0030354-b88] Mayford M, Bach ME, Huang YY, Wang L, Hawkins RD (1996). Control of memory formation through regulated expression of a CaMKII transgene. Science.

[pbio-0030354-b89] Shimshek DR, Kim J, Hubner MR, Spergel DJ, Buchholz F (2002). Codon-improved Cre recombinase (iCre) expression in the mouse. Genesis.

[pbio-0030354-b90] Reisel D, Bannerman DM, Schmitt WB, Deacon RM, Flint J (2002). Spatial memory dissociations in mice lacking GluR1. Nat Neurosci.

[pbio-0030354-b91] Suchanek B, Seeburg PH, Sprengel R (1997). Tissue specific control regions of the N-methyl-D-aspartate receptor subunit NR2C promoter. Biol Chem.

[pbio-0030354-b92] Kim J (2001). Improvement and establishment of the tTA-dependent inducible system in the mouse brain [dissertation].

